# Congenital absence of pericardium: two cases and a comprehensive review of the literature

**DOI:** 10.1259/bjrcr.20180117

**Published:** 2019-02-14

**Authors:** Pietro Sergio, Erika Bertella, Margherita Muri, Ilaria Zangrandi, Paolo Ceruti, Franco Fumagalli, Giancarlo Bosio

**Affiliations:** 1 Azienda Ospedaliera di Cremona, Cremona, Italy; 2 Spedali Civili di Brescia, Brescia, Italy

## Abstract

Congenital absence of pericardium (CAP) is a rare condition, generally asymptomatic or paucisymptomatic, nevertheless sporadic cases complicated by sudden death are described.

CAP can be diagnosed by CT and MRI. It is classified as total or partial, and partial defects are divided into left defects and right defects.

Interestingly, several articles highlight the correlation between CAP and some anatomical lung abnormalities, such as presence of lung parenchyma between the main pulmonary artery and ascending aorta, lung parenchyma between the base of the heart and left hemidiaphragm, and lung parenchyma between the proximal ascending aorta and right pulmonary artery.

Congenital absence of pericardium (CAP) is a rare condition, generally asymptomatic or paucisymptomatic, nevertheless sporadic cases complicated by sudden death are described.

CAP is usually discovered incidentally during cardiothoracic surgery or autopsy, and it has a prevalence of approximately one in 12,000 people.

A variety of cardiac and extracardiac malformations can also be associated with pericardial defects.

CAP can be diagnosed by CT and MRI. Absence of the pericardium is classified as total or partial, and partial defects are divided into left defects, which are more common, and right defects. Total defects are of little clinical importance, whereas partial defects, though rarely, can cause sudden death, and therefore the detection of this malformation is clinically important. Interestingly, several articles highlight the correlation between CAP and some anatomical lung abnormalities, such as presence of lung parenchyma between the main pulmonary artery and ascending aorta, lung parenchyma between the base of the heart and left hemidiaphragm, and lung parenchyma between the proximal ascending aorta and right pulmonary artery.

## Case presentation

**Figure 1.**
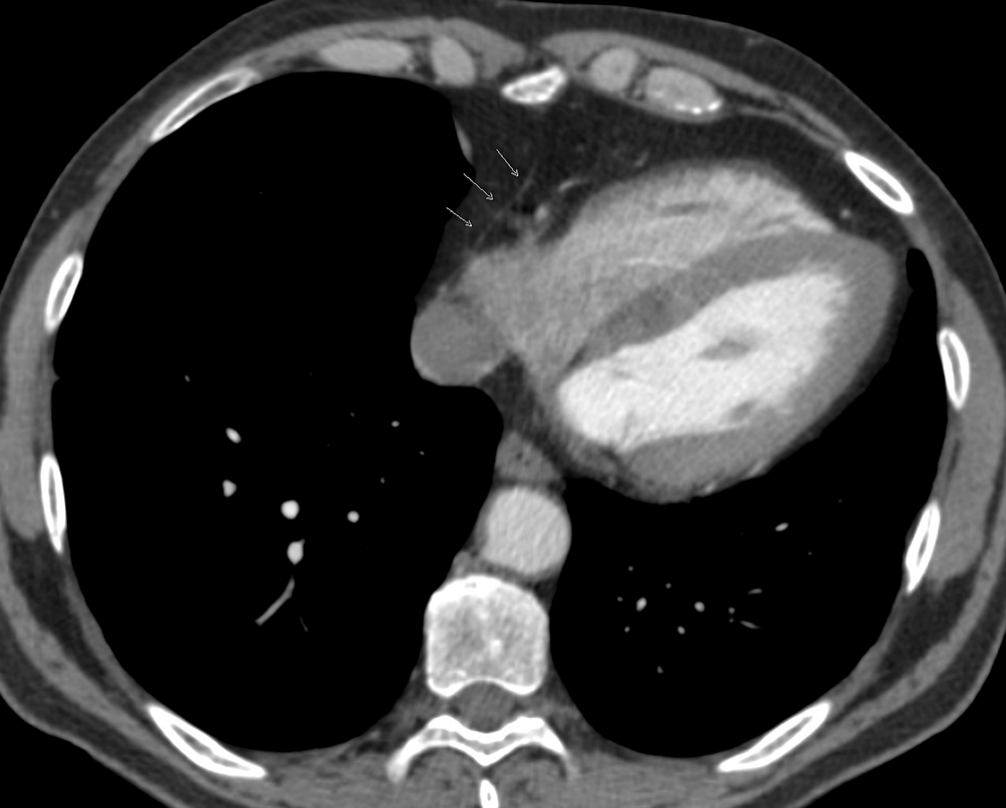
The pericardium is partially covering the right cardiac chambers and is absent over the left ventricle (indicated by arrows).

**Figure 2.**
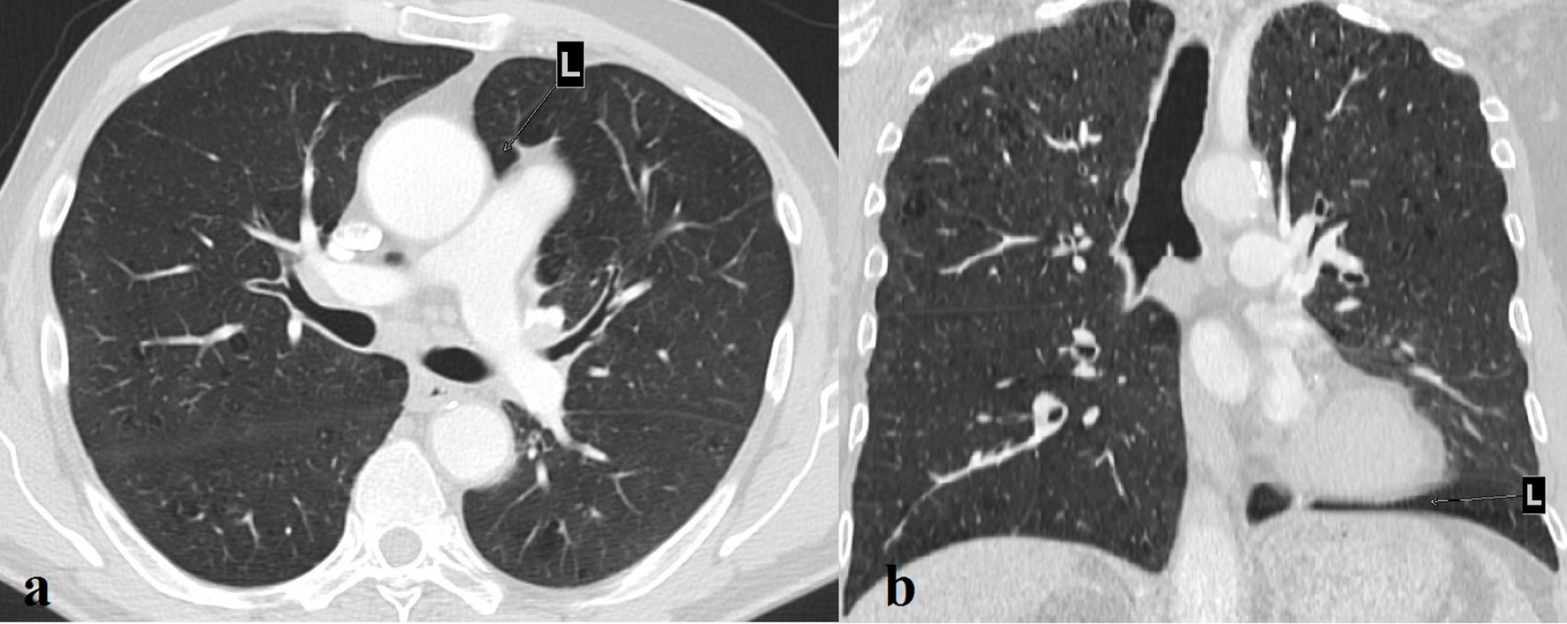
(a). Interposition of lung tissue between the aorta and main pulmonary artery (L). (b). Interposition of lung tissue between the diaphragm and the base of the heart (L).

The first case is a 66-year-old male, presented to the Emergency Department of Azienda Ospedaliera di Cremona complaining of atypical chest pain and palpitations. His medical past history was unremarkable. ECG revealed right axis deviation, a right bundle branch block, and no ST elevation. The troponin I value was mildly elevated, 40.1 ng l^−1^ (normal range: 0–34.2 ng l^−1^). We suspected an acute coronary syndrome, the patient was underwent to cardiac catheterization, which revealed no obstructive coronary artery disease of the left main coronary, but the right coronary artery was not detected. In order to exclude an aortic dissection or an anomalous origin of the right coronary, a CT angiography was carried out. CT showed a marked displacement of the heart in the left hemithorax. The pericardium was only partially visualized (Figure 1), lung tissue was evident between the ascending aorta and the main pulmonary artery, and between the base of the heart and the left hemidiaphragm (Figure 2a and b). The findings were considered signs of congenital absence of the pericardium.

**Figure 3.**
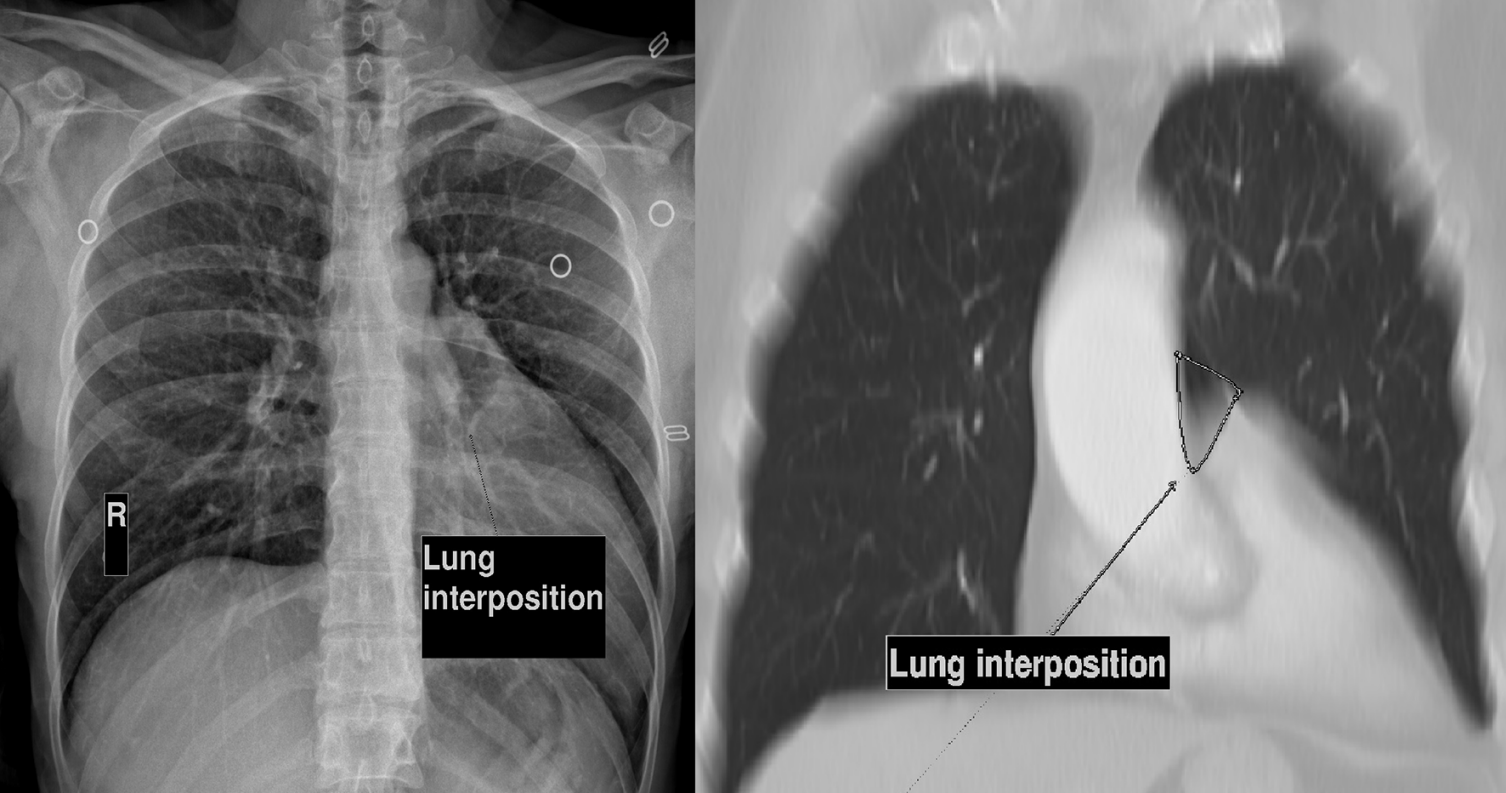
(a). Chest X-ray shows leftward position of the heart (Snoopy sign) and a hyperlucency between the aorta and main pulmonary artery (b). MIP reconstruction documents lung between the aorta and main pulmonary artery, region commonly lung-free. MIP, maximum intensity projection.

The second patient was a 49-year-old female, brought to the Emergency Department because of a road accident. Her chest X-ray was interesting, showing leftward position of the heart, and hyperlucency between the aorta and main pulmonary artery due to interposition of lung (Figure 3). The review of a previous chest CT showed a left pericardium defect.

## Discussion

Congenital absence of the pericardium is an uncommon cardiac defect estimated to occur in 1 in 10,000–14,000 people, and is usually discovered incidentally, *e.g.* during operations, autopsies and radiological studies.^1–4^

Most patients are asymptomatic, but atypical chest pain, dyspnoea and palpitations are common symptoms.^1–3,5^ Patients with CAP have a life expectancy comparable to that of the general population, but life-threatening complications, such as incarceration of left atrial appendage, ventricular herniation and torsion of the great vessels are described in the literature.^3,6^

Up to 30% of pericardial defects are associated with other cardiac abnormalities, including bicuspid aortic valve, mitral valve stenosis or prolapse, tetralogy of Fallot, atrial septal defect, or persistent ductus arteriosus.^2,3,5,6^ In addition, a variety of extracardiac malformations can also be associated with pericardial defects. These abnormalities include bronchogenic cysts, pulmonary sequestration, VACTER syndrome, diaphragmatic hernia, or hepatic hemangioendothelioma.^2,3,5,6^

Six categories of pericardial defects have been reported: total absence, right-sided defects (complete or partial), left-sided defects (complete or partial), and diaphragmatic defects.^6^

Left-sided defects are the most commonly reported, involving up to 70–80% of cases of pericardial defects, whereas right-sided pericardial absence is much rarer than left-sided defects and was reported in 4–17% of cases. Finally, total absence of the pericardium is the rarest of all defects, seen in only 9% of cases reported in the literature.^3,6^

In case of absent left pericardium, CT typically shows some indirect signs, such as abnormal presence of lung parenchyma in the usually lung-free aortopulmonary space and interposition of lung tissue between the left hemidiaphragm and the base of the heart.^1–3,7–9^

A direct sign of left CAP is represented by blocked view of the left pericardium.

In case of absent right pericardium, CT may show, as indirect sign, an abnormal presence of lung parenchyma in the space, usually lung-free, between the ascending aorta and the right pulmonary artery, and an anomalous cardiac contour due to right appendage, atrium or ventricle herniation.^3,5,10–12^ A direct sign of right CAP is represented by a blocked view of the right pericardium.

In case of a total absence of the pericardium, a combination of the previously described indirect signs may appear.^3^ A marked displacement of the heart into the left hemithorax may be present in total and left CAP.

Total defects are of little clinical importance, but partial defects, although rarely, may be the cause of sudden death, therefore the detection of this malformation is clinically important, and the recognition of the pulmonary anatomical abnormalities related to CAP may be useful clues to suggest and support the diagnosis.

We compared the most common described lung abnormalities associated with CAP (Table 1) with data from our experience (Table 2). We detected 1 case of left absence of pericardium in 12,288 individuals examined with chest CT.

**Table 1.** Indirect signs of CAP (Medline search)

Indirect signs of CAP (Medline search)
✓Lung parenchyma between the main pulmonary artery and ascending aorta, associated with partial (left) CAP and total CAP ✓Lung parenchyma between the base of the heart and left hemidiaphragm, associated with partial (left) CAP and total CAP ✓Lung parenchyma between the proximal ascending aorta and right pulmonary artery, associated with partial (right) CAP and total CAP

CAP, congenital absence of pericardium.

**Table 2.** Indirect signs of CAP: frequency from our data

Indirect signs of CAP: frequency from our data
Lung parenchyma between the main pulmonary artery and ascending aorta: 2 in 1,2888 cases, 1 case of partial left CAP ^a^ 1 case of post-procedural event, without CAP
Lung parenchyma between the base of the heart and left hemidiaphragm: 10 in 1,2888 cases, 1 case of partial left CAP ^a^ 9 cases with dorsal kyphosis, causing heart displacement with lung interposition between heart and left hemidiaphragm, without CAP
Lung parenchyma between the proximal ascending aorta and right pulmonary artery: 0 in 1,2888 cases

CAP, congenital absence of pericardium.

a Same patient showing left CAP, with the evidence of both lung between the main pulmonary artery and ascending aorta, and lung between the base of the heart and left hemidiaphragm.

**Figure 4.**
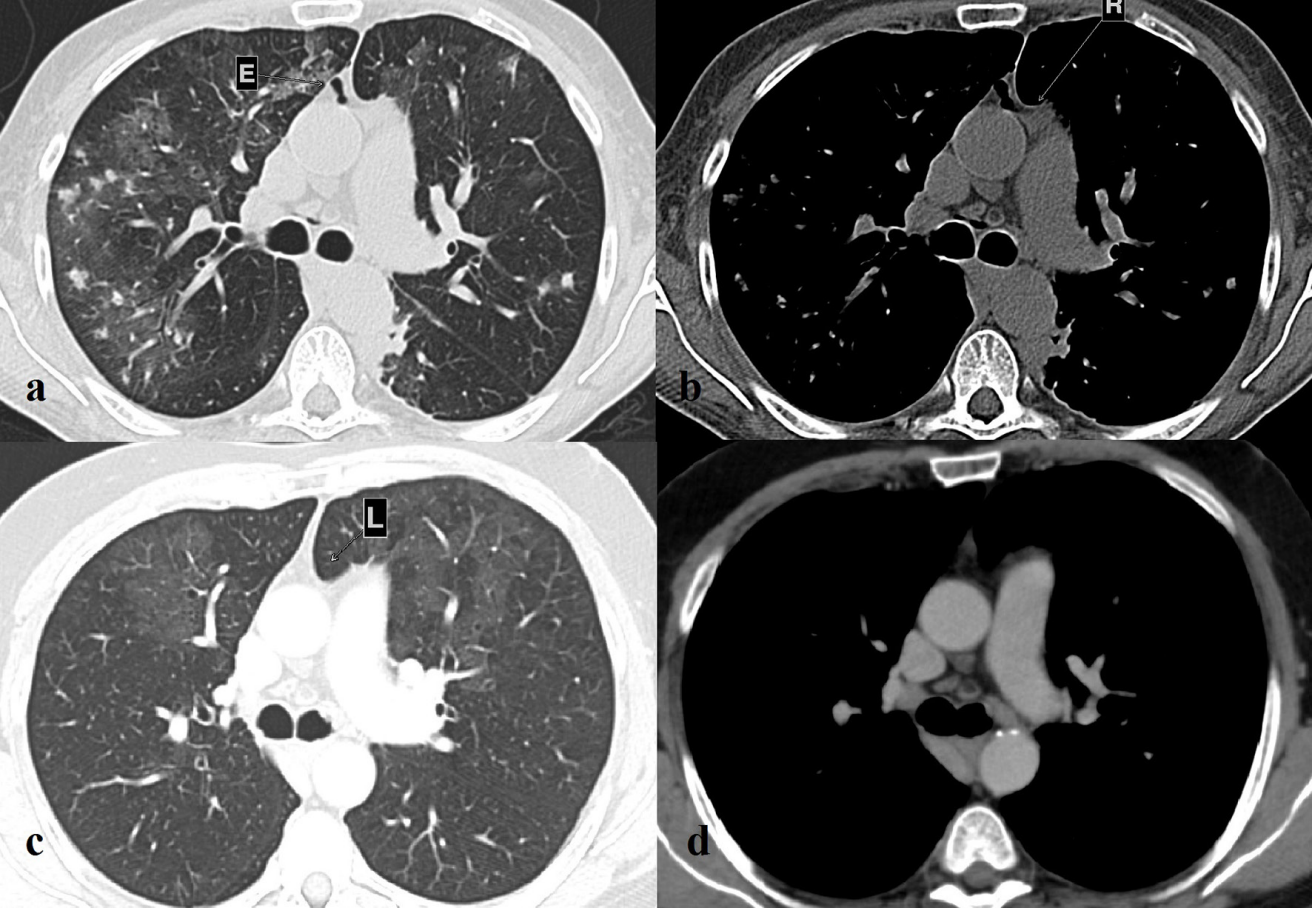
(a). Lung window: a moderate mediastinal emphysema (E) is evident. (b). Mediastinal window: the axial image shows the pre-aortic recess (R) slightly filled with fluid. In addition, the region between the ascending aorta and main pulmonary artery shows a blunt morphology. (c). Lung window: CT performed 2 years later shows interval changes of the region between the ascending aorta and main pulmonary artery. A small amount of lung interposition between the aorta and pulmonary artery (L) is evident, mimicking a condition of CAP. (d). Mediastinal window: the aortopulmonary junction shows a sharp morphology. CAP, congenital absence of pericardium.

Moreover*,* we identified two cases with lung interposition between the ascending aorta and main pulmonary artery, one case characterized by CAP, specifically a left defect (Figures 1, 2a and b). The other case involved a patient with no pericardial defect and with a history of mediastinal emphysema caused by complications that arose during the insertion of a right internal jugular central venous catheter (Figure 4). At the time of insertion of the central venous catheter, the presence of the aortopulmonary pericardial recess was evident (Figure 4a and b), but no longer clearly evident at the CT check about 2 years later (Figure 4c and d). We speculated on the possible cause of the pericardial recess changes, considering the hypothesis that the post-procedural mediastinal emphysema could have caused a regional fat necrosis, and we also hypothesize that major thoracic traumas might cause similar anomalies.^11^

In our experience, no case with interposition of pulmonary parenchyma between the ascending aorta and right pulmonary artery was detected.

**Figure 5.**
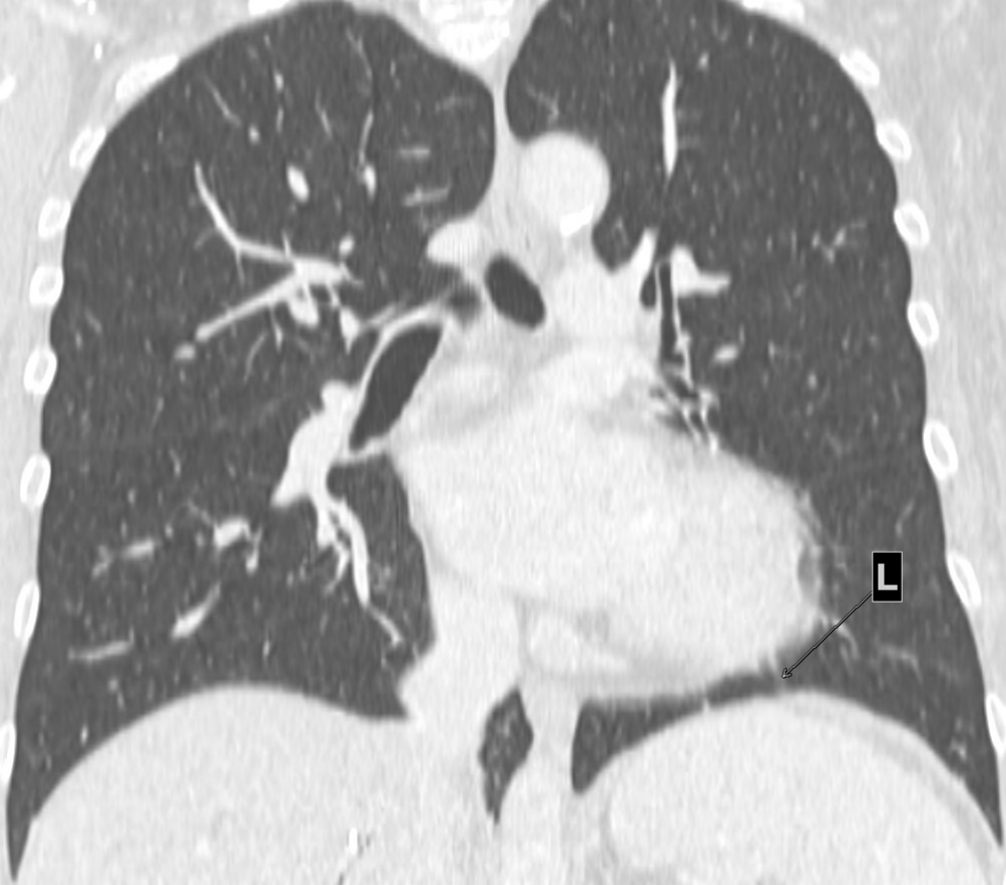
The image shows interposition of pulmonary parenchyma between the base of the heart and left hemidiaphragm (L). The patient does not have congenital nor acquired pericardial defect.

Finally, the presence of pulmonary parenchyma between the inferior side of the heart and the left hemidiaphragm was found in several patients, one with left absence of pericardium (Figures 1, 2a and b), and all the others characterized by dorsal kyphosis (Figure 5), cause of displacement of the heart into the left hemithorax and interposition of pulmonary parenchyma between the base of the heart and left hemidiaphragm. In conclusion, we suggest taking into consideration the lung anomalies associated with CAP, although not exclusive of this condition, to support its diagnosis, and to properly recognize a total or partial defect, because of the potential complications in partial defects.

## Learning points

1. Congenital absence of pericardium is a rare condition, usually discovered incidentally. This entity can be diagnosed by CT and MRI.
2. It is widely known the correlation between congenital absence of pericardium and some anatomical lung abnormalities, such as:
  - lung parenchyma between the main pulmonary artery and ascending aorta,
  - lung parenchyma between the base of the heart and left hemidiaphragm,
  - lung parenchyma between the proximal ascending aorta and right pulmonary artery.
3. It is useful taking into consideration these lung anomalies to support the diagnosis of congenital absence of pericardium, and to properly recognize a total or partial defect, because of the potential complications in partial defects.

## References

[1] Macaione F, Barison A, Pescetelli I, Pali F, Pizzino F, Terrizzi A, et al Quantitative criteria for the diagnosis of the congenital absence of pericardium by cardiac magnetic resonance. Eur J Radiol 2016; 85: 616–24. doi: 10.1016/j.ejrad.2015.12.021 26860675

[2] Steinberg C, Pelletier MJ, Perron J, Kumar A, Champagne J Sudden cardiac arrest due to subtotal absence of left-sided pericardium--case report and review of the literature. Congenit Heart Dis 2013; 8: E92–E98. doi: 10.1111/j.1747-0803.2012.00686.x 22698265

[3] Shah AB, Kronzon I Congenital defects of the pericardium: a review. Eur Heart J Cardiovasc Imaging 2015; 16: 821–7. doi: 10.1093/ehjci/jev119 26003149

[4] Hiraoka K, Yamazaki S, Hosokawa M, Suzuki Y Bronchogenic cyst associated with congenital absence of the pericardium. J Surg Case Rep 2015; 2015: rjv052: 23 04 2015. doi: 10.1093/jscr/rjv052 25907540 PMC4407419

[5] Palau P, Domínguez E, García-González P, Gallego J, Bosch MJ, Sieso E, et al Isolated partial congenital absence of the pericardium: a familial presentation. Can J Cardiol 2016; 32: 1039.e1–1039.e2. doi: 10.1016/j.cjca.2015.09.001 26774230

[6] Verde F, Johnson PT, Jha S, Fishman EK, Zimmerman SL Congenital absence of the pericardium and its mimics. J Cardiovasc Comput Tomogr 2013; 7: 11–17. doi: 10.1016/j.jcct.2013.01.003 23452995

[7] Garnier F, Eicher JC, Philip JL, Lalande A, Bieber H, Voute MF, et al Congenital complete absence of the left pericardium: a rare cause of chest pain or pseudo-right heart overload. Clin Cardiol 2010; 33: E52–E57. doi: 10.1002/clc.20607 20043342 PMC6653773

[8] Jurko A, Minarik M, Cisarikova V, Polacek H, Schusterova I Congenital complete and partial absence of the left pericardium. Wien Med Wochenschr 2013; 163(17-18): 426–8. doi: 10.1007/s10354-013-0178-4 23381230

[9] Psychidis-Papakyritsis P, de Roos A, Kroft LJ Functional MRI of congenital absence of the pericardium. AJR Am J Roentgenol 2007; 189: W312–W314. doi: 10.2214/AJR.05.1655 18029841

[10] Ono S, Ichikawa T, Iino M, Yamada Y, Sekiguchi T, Nakagawa T, et al Erratum to: congenital pericardial defect: a case of right pericardial partial absence with normal parietal pleura. Jpn J Radiol 2015; 33: 157. doi: 10.1007/s11604-015-0401-x 25687938

[11] Koo CW, Newburg A Congenital absence of the right pericardium: embryology and imaging. J Clin Imaging Sci 2015; 5: 12. doi: 10.4103/2156-7514.152338 25861546 PMC4374196

[12] Rajiah P, Kanne JP Computed tomography of the pericardium and pericardial disease. J Cardiovasc Comput Tomogr 2010; 4: 3–18. doi: 10.1016/j.jcct.2010.01.004 20159622

